# Interleukin-36 Is Highly Expressed in Skin Biopsies from Two Patients with Netherton Syndrome

**DOI:** 10.3390/dermatopathology11030024

**Published:** 2024-08-12

**Authors:** Johannes Pawlowski, Tatsiana Pukhalskaya, Kelly Cordoro, Marina Kristy Ibraheim, Jeffrey P. North

**Affiliations:** 1 Department of Dermatology, University Hospital Mainz, 55131 Mainz, Germany; johannes.pawlowski@unimedizin-mainz.de; 2 Department of Dermatology and Pathology, University of California San Francisco, San Francisco, CA 94143, USA; 3 Department of Dermatology and Pediatrics, University of California San Francisco, San Francisco, CA 94143, USA; kelly.cordoro@ucsf.edu; 4 Department of Dermatology, Loma Linda University, Loma Linda, CA 92354, USA; mibraheim@llu.edu

**Keywords:** interleukin-36, Netherton syndrome, SPINK5, LEKTI

## Abstract

Netherton syndrome (NS) is a rare autosomal recessive disorder that occurs due to a loss-of-function mutation in SPINK5; this loss results in significant inflammation, as well as perturbations of the skin barrier’s integrity and functionality. While it is unclear which inflammatory pathways contribute to the development of NS, recent studies have demonstrated the expression of interleukin (IL)-17/IL-36, as well as several Th2 cytokines. Consequently, immunohistochemistry (IHC) with IL-36 may serve as a potential tool for aiding the histopathological diagnosis of this condition. In this case series, we present two cases of NS and capture their immunostaining pattern with IL-36. Both cases demonstrated robust expression of IL-36. This finding bolsters the hypothesis that NS is partially driven by Th17 activation and suggests the potential utility of IL-36 IHC as part of the workup for this rare and diagnostically elusive entity. LEKTI IHC was negative in one biopsy, revealing a limitation of this stain in diagnosing NS.

## 1. Introduction

Netherton syndrome (NS) is a rare autosomal recessive disorder resulting from loss-of-function mutations in the SPINK5 gene, a gene encoding the serine protease inhibitor of Kazal-type 4 (LEKTI). Loss of this protein greatly impairs the skin barrier by engendering excessive desquamation, transepidermal water loss, and inflammation [1]. The disease presents classically with congenital ichthyosis, trichorrhexis invaginata, atopy, and elevated serum immunoglobulin (Ig)E [2]. While the clinical spectrum of disease is broad, NS phenotypes may be clustered as ichthyosis linearis circumflexa (NS-ILC) and scaly erythroderma (NS-SE) [3]. Depending on disease severity, NS can precipitate life-threatening sequelae in infants, including dehydration, electrolyte disturbances, seizures, failure to thrive, and recurrent infections [4].

The diagnosis of NS is often made when clinical features trigger suspicion and genetic testing is obtained for confirmation. Given the variety of cutaneous manifestations of NS, diagnosis is often delayed for years. Skin biopsy provides an additional data point for assessment. Biopsy specimens from NS patients can show psoriasiform epidermal hyperplasia, parakeratosis, elongated rete ridges, and a diminished granular layer. Microabscesses and dilated blood vessels may also be found in the epidermis and superficial dermis, respectively [5]. Spongiosis, the presence of eosinophils, and dyskeratotic keratinocytes, have also been documented [5].

These histopathologic findings overlap with psoriasis, which also features regular acanthosis, parakeratosis with neutrophils, hypogranulosis, and ectatic blood vessels in the papillary dermis [6,7,8]. Psoriasis is an inflammatory dermatosis promulgated by several inflammatory pathways, including T-helper (Th)1 cells, Th17/Th22 cells, and interleukin (IL)-23 [9]. Activation of the Th17 pathway increases the cutaneous production of IL-36, a pro-inflammatory cytokine that is highly expressed in psoriatic skin compared to spongiotic dermatitis [6,10]. Recent studies exploring the utility of IL-36 immunohistochemistry (IHC) have demonstrated the consistent diffusely strong expression of IL-36 in psoriatic skin compared to other psoriasiform dermatoses and eczema [6,7,8].

Given recent molecular studies identifying IL-17/IL-36 activity in NS, we aimed to assess IL-36 expression in lesional skin of NS patients [3,11]. We identified two cases of NS with the NS-ILC phenotype from our respective institutions based on molecular confirmation of SPINK5 mutation. Formalin-fixed, paraffin-embedded specimens were stained with hematoxylin–eosin and IL-36 (IL-36 gamma clone 2F4, 1:300 dilution, catalog # ab1456786; AbCam, Cambridge, MA, 4 μm sections). The grading of IL-36 was assessed based on cytoplasmic expression in the upper epidermis and adhered to the following 0–4 scoring system previously described in the literature: 0, negative; 1, focal weak; 2, diffuse weak; 3, focal, strong; 4, diffuse strong. A total of 6–8 cases were reviewed by two board-certified dermatopathologists (J.N. and T.P.).

## 2. Case Descriptions

### 2.1. Case 1

**Figure 1:**
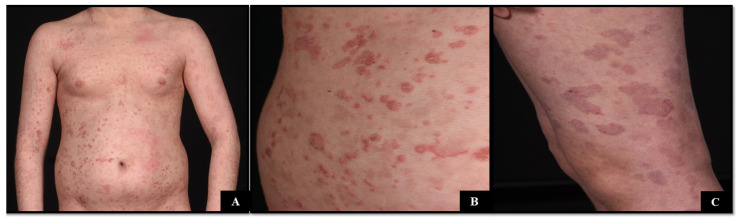
(**A**–**C**) Oval and annular erythematous macules, patches, and plaques with slight scale affecting the trunk and extremities.

A 15-year-old boy with a history of NS [SPINK5 mutation, p.(Gln52lysfs*5); p.(Cys297)], allergic rhinitis, allergic conjunctivitis, and food allergies presented to the dermatology clinic with a several-year history of rash that transiently improved with methotrexate, cyclosporine, and dupilumab. A skin exam demonstrated coin-shaped, somewhat serpiginous erythematous plaques with a double collar of scale (Figure 1A–C). Trichoscopy revealed a portion of the proximal hair strand invaginated into the distal portion, consistent with trichorrhexis invaginata. Serologic testing demonstrated elevated serum IgE (5251 kU/L; reference > 20 kU/L).

**Figure 2:**
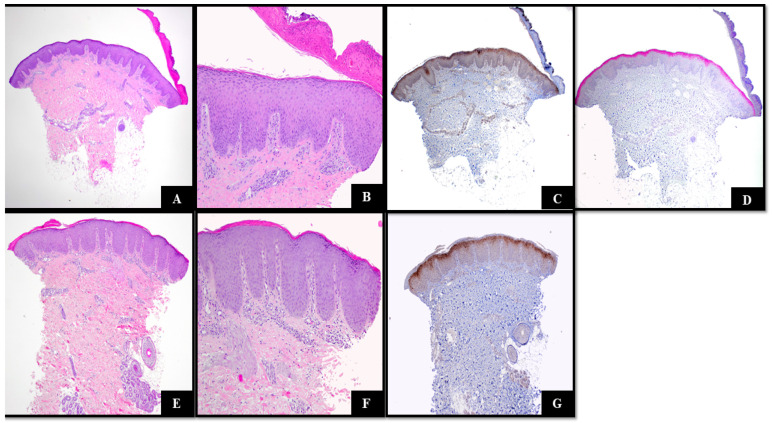
(**A**,**E**) Hematoxylin and eosin (H&E)-stained sections demonstrating psoriasiform epidermal hyperplasia, parakeratosis with variable hypergranulosis. Mildly dilated blood vessels and a superficial perivascular lymphocytic infiltrate were also present. (H&E 40×); (**B**,**F**) features at high magnification (H&E 100×); (**C**,**G**) IL-36 staining grade 4 (IL-36 40×); (**D**) LEKTI strongly staining the granular layer (LEKTI 40×).

Two punch biopsies were performed: one before the initiation of systemic therapy (Figure 2A–D) and one after the initiation of dupilumab (Figure 2E–G). Both biopsies revealed psoriasiform epidermal hyperplasia, mild spongiosis, parakeratosis with variable serum accumulation, and a normal to slightly increased granular layer. Additionally, a sparse superficial perivascular lymphocytic infiltrate and mildly dilated vessels in the superficial vascular plexus were noted (Figure 2A,B,E,F). Slight spongiosis was noted, but no dyskeratosis was observed. Sparse eosinophils were present. Both specimens demonstrated diffuse, high-intensity expression of IL-36 in the upper epidermis (grade 4 positivity) (Figure 2C,G). Immunohistochemical studies revealed the strong expression of LEKTI in the stratum corneum (Figure 2D).

### 2.2. Case 2

**Figure 3:**
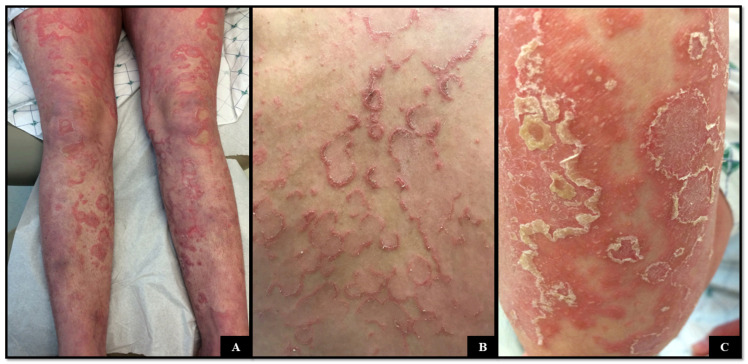
(**A**–**C**) Numerous erythematous, serpiginous plaques with a double edge scale on the trunk and extremities.

A 24-year-old woman with a history of a peanut allergy and NS [SPINK 5 mutation, HET c.C891T (+10In-Ex), p.K824Rfs*117] presented for evaluation of a new flare of her skin disease. The patient’s symptoms began in infancy with erythroderma prompting an initial diagnosis of atopic dermatitis. After developing a pustular component to her skin disease, a revised diagnosis of psoriasis was rendered. Two biopsies performed in childhood demonstrated psoriasiform dermatitis and subcorneal pustular dermatitis. The patient later developed erythematous, serpiginous plaques with a double-edged scale (Figure 3A,B); an eyebrow hair mount demonstrated trichorrhexis invaginata. Serologic testing demonstrated elevated serum IgE (8110 kU/L). The patient attempted numerous therapies including intravenous immunoglobulin (IVIG), adalimumab, secukinumab, and dupilumab, of which IVIG resulted in the greatest improvement.

**Figure 4:**
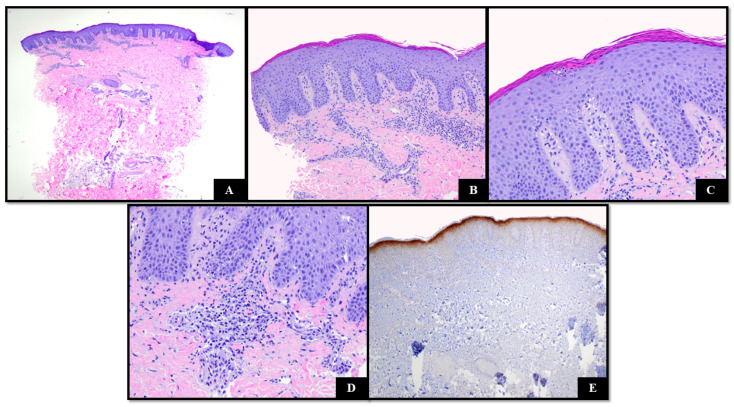
(**A**) H&E demonstrating regular acanthosis, broad parakeratosis with focal neutrophilic inflammation, mild spongiosis, and superficial perivascular inflammation comprising lymphocytes. (H&E 40×); (**B**–**D**) features at higher magnification ((**B**) H&E 100×, (**C**,**D**): H&E 400×); (**E**) IL-36 staining grade 4 (IL-36 40×).

A punch biopsy of a plaque on the thigh during a flare demonstrated acanthosis, broad parakeratosis with hypogranulosis, mild spongiosis, a tiny focus of neutrophils in the upper spinous zone, and superficial perivascular lymphocytic inflammation (Figure 4A–D). No dyskeratosis was noted. Spare eosinophils were present. The specimen strongly and diffusely expressed IL-36 in the upper epidermis, indicating grade 4 expression (Figure 4E).

## 3. Discussion

This case series captures the histopathologic findings and IL-36 immunostaining patterns of two patients with NS. In both cases, the affected skin highly expressed IL-36, suggesting that Th17 activation contributes at least partially to the pathogenesis of NS [3,11]. LEKTI, the protein product of the SPINK5 gene, is a serine protease inhibitor produced by keratinocytes between the granular layer and stratum corneum [12,13,14,15]. LEKTI inhibits several serine proteases found in this portion of the epidermis, including kallikrein-related proteinases (KLK) 5, KLK7, KLK14, and epidermal elastase 2; this counterbalance allows for appropriate rates of desquamation. In the absence of LEKTI, these serine proteases function unfettered, accelerating cadherin and desmosome degradation, altering pro-filaggrin processing, and triggering the premature separation of keratinocytes in the upper epidermis [15,16,17,18,19,20]. Hyperactivity of KLK5 upregulates protease-activated receptor-2, triggering the expression of numerous pro-inflammatory cytokines such as thymic stromal lymphopoietin (TSLP), CXCL8, TNF-α, and IL-17. This combination results in significant skin barrier impairment [11,16,17,21,22,23,24,25]. Interestingly, TSLP and IL-17/TNF-α are associated with Th2 and Th17 responses, respectively [3,11,15,22,26].

The activation of both Th2 and Th17 may explain the clinical and histologic findings seen in NS. The atopic features seen in NS and spongiosis on histopathology may be promulgated by the Th2 axis [5,26,27]. Activation of Th17 drives the production of IL-17 and TNF-α, both of which potentiate the production of IL-36 by keratinocytes. This generates a positive feedback loop, as IL-36 promotes further activity of Th17 [28]. Increased IL-36 activity is associated with aberrant cornification and excessive epidermal proliferation [29,30,31,32]. In keratinocytes, both IL-17 and IL-36 cytokines prompt the expression and secretion of CCL20 and CXCL8, further instigating an inflammatory response and attracting neutrophils [33,34]. This inflammatory pathway is a key driver of psoriasis, and previous studies have illustrated the association between IL-36 IHC expression and psoriasis [6,7,8].

The psoriasiform changes observed in NS, as well as the strong IL-36 expression seen in this case series, may be explained by this inflammatory cascade. Previously, a large case series captured the histopathological features found in NS. These included psoriasiform epidermal hyperplasia and, less frequently, parakeratosis, stratum corneum splitting, apoptotic keratinocytes, granulocytic dermal infiltrate, and dilated vessels in the papillary dermis [5]. The specimens in our case series demonstrated similar findings, with psoriasiform hyperplasia as the most prominent feature. Spongiosis has been documented in NS and was observed in varying degrees in the biopsies in our series, likely due to Th2 activation [5,27]. Both clinically and histopathologically, NS may be difficult to distinguish from eczema. In such cases, IL-36 expression may serve as an ancillary test to distinguish between these states. Additionally, in our first case of NS, LEKTI expression with IHC was observed despite a confirmed SPINK5 mutation. This finding contrasts with a previous study reporting LEKTI antibody’s sensitivity and specificity as 100% [35]. While this scenario may occur rarely, the use of IL-36 immunostain may provide an additional data point for use by clinicians.

While the diagnosis of NS poses its own challenges, its treatment remains equally elusive. No targeted therapy for NS exists. Given the atopic features seen in NS, some have attempted treatment with dupilumab to target the Th2 pathway. This approach has demonstrated improvement in pruritus in some patients, though the response may only be transient [26,27,36,37,38]. Other case reports have detailed clinical improvement with biologic therapies targeting both IL-17 and IL-23 in some patients [26,39,40,41,42]. It appears that the loss of LEKTI can result in the activation of multiple inflammatory pathways including both the Th17 and Th2 pathways. Future studies exploring the long-term efficacy of these agents and others that inhibit other inflammatory pathways involved in NS are needed, but Th17 activation in NS supports the potential therapeutic benefit of agents that target cytokines in these pathways [11].

## 4. Conclusions

Data from this small case series further support the assertion that NS is, at least in part, driven by Th17 inflammation. The use of IL-36 immunostaining could serve as a helpful diagnostic tool, as it can help to distinguish NS from some inflammatory dermatoses such as eczematous conditions like atopic dermatitis that are primarily driven by Th2 inflammation. However, IL-36 staining does not distinguish NS from other inflammatory diseases with Th17 activation such as psoriasis. Furthermore, LEKTI staining on biopsy specimens may not always show the loss of LEKTI expression in biopsies of NS. Positive IL-36 expression detected by immunostaining may serve as an additional clue raising suspicion for the diagnosis of NS.

## Data Availability

Data pertaining to the cases are presented in this article. Further inquiries can be directed to the corresponding author.
